# Viewpoint on ‘Oscillating paramagnetic Meissner effect and Berezinskii–Kosterlitz–Thouless transition in underdoped Bi_2_Sr_2_CaCu_2_O_8+δ_’

**DOI:** 10.1093/nsr/nwae376

**Published:** 2024-10-26

**Authors:** Egor Babaev

**Affiliations:** Department of Physics, KTH Royal Institute of Technology, Sweden

## Abstract

The experimental team achieves impressive progress in detecting the signature of the local magnetic response of a system close to a phase transition.

The Nobel-prize-winning works by Kostelitz and Thouless [1,2] and by Berezinskii [3,4] established the new paradigm of phase transitions in planar systems that break continuum symmetry. In contrast to the Landau paradigm, these transitions are not associated with breaking a symmetry, but are rather associated with the transition to a state of topological (algebraic) order. The existence of such order depends on whether a system has free topological excitations: in the original Berezinskii–Kosterlitz–Thouless picture, these were single-quantum vortices in superfluids. Above the superfluid phase transition, these vortices are free, and below the transition, these vortices are bound into vortex-antivortex pairs [5]. Related ideas of vortex-driven phase transitions date back to the discussion of phase transitions in three-dimensional systems. Onsager [6] proposed that the superfluid phase transition is governed by the proliferation of vortex loops; similar ideas were explored by Feynman. Dasgupta and Halperin developed a vortex-loop-based picture of the phase transition in superconductors [7]. The vortex-driven transitions were studied in Monte Carlo simulations of superconducting models [8].

In principle, a phase transition is associated with the onset of long- and quasi-long-range orders, which are traditionally measured by the global response of the system, such as the system’s transport properties. However, especially in light of recent developments, experimental techniques that can visualize the system locally and directly probe the nature of the topological excitations that drive a phase transition are crucially needed.

This is a highly nontrivial task experimentally, even for the most sensitive techniques, such as scanning superconducting quantum interference device (SQUID) experiments. For example, the Berezinskii–Kosteritz–Thouless transition happens at the temperature where the free energy of the vortex vanishes, and hence vortices proliferate. Interesting crossovers with dilute vortex excitations could also happen at finite but large-length scales for layered systems. In a dimensional picture of vortex loops, the vortex loops form extremely dense overlapping tangles [8], which makes resolution of what happens at short-length scales even more challenging. Further challenges for the scanning SQUID technique are associated with the long magnetic field penetration length near a resistive phase transition in addition to vortex mobility and the presence of flux in both directions. For that reason, a large part of scanning SQUID microscopy works are focused on resolving individual vortices at low temperatures far below phase transitions. At the same time, the quality of available two-dimensional or quasi-two-dimensional samples grows, which makes experimental studies that aim to distinguish thermal vortex excitations from other phenomena, such as intrinsic Josephson-junction-like physics, very timely and important.

Wang *et al.* [9] proposed using the local magnetic response measured by a nano-SQUID to indirectly probe the physics of the topological-defect-driven phase transition. The authors used scanning SQUID susceptometry to image the local magnetic response of the underdoped high Curie-temperature $(T_c)$ superconductor Bi$_{2}$Sr$_{2}$CaCu$_{2}$O$_{8+\delta }$ (Bi2212) in some of the most experimentally challenging regimes near the critical temperature.

The experimental team achieved impressive progress in detecting the signature of the local magnetic response of a system close to a phase transition. The study is important not only in understanding the physics of this material, but also in its significant potential implications well beyond the physics of Bi2212. It has great potential to open new horizons well beyond conventional vortex physics. Namely, in recent years, it has been realized that new states of matter can arise in systems with multiple species of topological defects [10], such as vortices carrying an arbitrary fraction of the magnetic flux quantum [11]. Phase transitions in such systems can lead to phases different from superconducting and normal states [12]; a crucially important contribution to the investigation of such materials could be local magnetometry, and hence even from that broader viewpoint, the impressing progress in magnetometry in regimes close to vortex-driven phase transitions developed in this work is crucially important.

## References

[1] Kosterlitz JM, Thouless DJ. J Phys C 1972; 5: L124. 10.1088/0022-3719/5/11/002

[2] Kosterlitz JM, Thouless DJ. J Phys C 1973; 6: 1181. 10.1088/0022-3719/6/7/010

[3] Berezinskii VL . Sov Phys JETP 1971; 32: 493–500.

[4] Berezinskii VL . Sov Phys JETP 1972; 34: 610.

[5] Kosterlitz JM . J Phys Condens Matter 2016; 28: 481001. 10.1088/0953-8984/28/48/481001 27665689

[6] Onsager L . Il Nuovo Cimento (1943–1954) 1949; 6: 279–87. 10.1007/BF02780991

[7] Dasgupta C, Halperin BI. Phys Rev Lett 1981; 47: 1556–60. 10.1103/PhysRevLett.47.1556

[8] Nguyen AK, Sudbø A. Phys Rev B 1998; 57: 3123–43. 10.1103/PhysRevB.57.3123

[9] Wang SY, Yu YJ, Hao JX et al. Natl Sci Rev 2024; 11: nwad249. 10.1093/nsr/nwad249 38577674 PMC10989300

[10] Svistunov B, Babaev E, Prokofev N. Superfluid States of Matter. Boca Raton, FL: CRC Press, 2015. 10.1201/b18346

[11] Iguchi Y, Shi RA, Kihou K et al. Science 2023; 380: 1244–7. 10.1126/science.abp9979 37262195

[12] Grinenko V, Weston D, Caglieris F et al. Nat Phys 2021; 17: 1254–9. 10.1038/s41567-021-01350-9

